# Analyzing the ‘Bradykinesia Complex’ in Parkinson's Disease

**DOI:** 10.1002/mds.70082

**Published:** 2025-10-17

**Authors:** Giulia Paparella, Martina De Riggi, Antonio Cannavacciuolo, Daniele Birreci, Davide Costa, Luca Angelini, Danilo Alunni Fegatelli, Alfonso Fasano, Alberto J. Espay, Matteo Bologna

**Affiliations:** ^1^ IRCCS Neuromed Pozzilli Italy; ^2^ Neurophysiopathology Unit, Department of Translational Biomedicine and Neuroscience, University of Bari Aldo Moro Bari Italy; ^3^ Department of Human Neurosciences Sapienza University of Rome Rome Italy; ^4^ Department of Life Sciences, Health and Health Professions Link Campus University Rome Italy; ^5^ Department of Biomedical Sciences Humanitas University Milan Italy; ^6^ IRCCS Humanitas Research Hospital Milan Italy; ^7^ James J. and Joan A. Gardner Family Center for Parkinson's Disease and Movement Disorders, Department of Neurology University of Cincinnati Cincinnati Ohio USA

**Keywords:** Parkinson's disease, bradykinesia, kinematic analysis, finger‐tapping, aging

## Abstract

**Background:**

Bradykinesia is the hallmark sign of parkinsonism. We recently proposed redefining bradykinesia as a complex of motor abnormalities, each reflecting separate pathophysiological elements.

**Objective:**

To analyze the ‘bradykinesia complex’ in Parkinson's disease (PD) and healthy elderly individuals.

**Methods:**

We conducted a finger‐tapping kinematic analysis in 350 individuals (192 PD patients OFF medication and 158 healthy controls). A subsample of 129 patients was also tested ON medication. Group comparisons were followed by unsupervised clustering. Receiver operating characteristic (ROC) analyses defined optimal kinematic cut‐offs to detect individual motor abnormalities. We then quantified the prevalence and combinations of these features per subject. Using Bayes' theorem, we estimated the probability of PD based on the observed combination of bradykinesia features. Regression analyses served to identify predictors of kinematic alterations.

**Results:**

Patients exhibited reduced velocity and amplitude as well as altered rhythm and sequence effect compared with controls (all *P‐*values < 0.001). Cluster analysis revealed substantial group overlap. ROC analyses showed that bradykinesia (movement slowness) was the most common and accurate feature for distinguishing PD, with its diagnostic power improving when combined with other motor abnormalities (hypokinesia, dysrhythmia, sequence effect). The likelihood of correctly identifying PD increased with the number of observed abnormalities, reaching up to 95% when all features were present. Levodopa improved motor performance, but the motor abnormality patterns remained unchanged.

**Conclusions:**

The detailed bradykinesia features assessment was crucial for differentiating PD individuals from controls. Diagnostic accuracy requires considering multiple motor abnormalities together, irrespective of the specific combination. Advancing our understanding of the ‘bradykinesia complex’ has clinical and pathophysiological implications. © 2025 The Author(s). *Movement Disorders* published by Wiley Periodicals LLC on behalf of International Parkinson and Movement Disorder Society.

Bradykinesia is the hallmark feature of parkinsonism and, according to current clinical criteria for Parkinson's disease (PD), is defined by movement slowness and the sequence effect (progressive reduction in speed and/or amplitude as movements are continued).^1^ We recently proposed redefining bradykinesia as a complex of motor abnormalities, each representing separate pathophysiological elements.^2^ We proposed describing the primary phenomenological features of the ‘bradykinesia complex’ in each subject, for example, bradykinesia (movement slowness), hypokinesia (reduced movement amplitude), dysrhythmia (altered movement rhythm through interruptions, accelerations, or halts), and sequence effect (reduction in speed and/or amplitude).^2^ Our proposal recommended the assessment of these individual features and their possible combination, hypothesizing that bradykinesia with sequence effect and/or additional motor abnormalities strongly suggests parkinsonism.^2^, ^3^ In contrast, isolated bradykinesia and related features may be nonspecific findings, occurring across various conditions, including non‐parkinsonian etiologies and healthy aging.^4^, ^5^, ^6^, ^7^, ^8^, ^9^, ^10^, ^11^, ^12^, ^13^, ^14^, ^15^, ^16^

Following our article, some studies have examined the ‘bradykinesia complex’ in different settings.^6^, ^15^, ^16^, ^17^, ^18^, ^19^, ^20^, ^21^, ^22^, ^23^, ^24^, ^25^, ^26^, ^27^, ^28^, ^29^, ^30^, ^31^ In a recent kinematic study, we assessed bradykinesia features in PD participants, individuals with essential tremor, and healthy controls (HCs).^6^ The study confirmed substantial overlap in kinematic profiles across groups, reinforcing the idea that certain motor features are not exclusive to PD.^10^, ^15^, ^16^, ^32^ The study also indicated bradykinesia as the best distinguishing characteristic between groups.^6^ Other studies assessing finger‐tapping using customized smartphone apps and typing tests highlighted the complexity of impaired movement execution in PD.^27^, ^28^ Again, a functional neuroimaging study applied the redefined bradykinesia framework to investigate the neural correlates of each motor abnormality, uncovering distinct pathophysiological mechanisms.^33^ Despite the contribution of the abovementioned studies, no previous reports have yet focused on how bradykinesia and related features specifically combine in individuals with PD and in healthy subjects. Understanding the combination of bradykinesia features may help provide phenomenological insights useful in routine clinical assessment and in accurate clinical classification.^2^, ^3^, ^34^

In this article, we objectively investigate the ‘bradykinesia complex’^2^ in a large sample of PD participants and in healthy elderly individuals. We used a kinematic analysis system to assess the finger‐tapping maneuver (ie, the most widely used task in clinical settings).^1^, ^3^, ^4^, ^6^, ^29^, ^35^, ^36^ We first conducted group comparisons between patients and HCs on kinematic variables to identify overall motor differences. Next, we applied unsupervised clustering to explore the underlying structure of the data independently of clinical labels. This analysis revealed a substantial overlap between groups, highlighting the heterogeneity of motor impairments and the limitations of binary classification. To further evaluate the discriminative power of each kinematic variable, we performed receiver operating characteristic (ROC) curve analyses and identified optimal diagnostic cut‐off values. Then, we examined how the different features combine in individual cases and we applied Bayesian classification to quantify the probability of PD diagnosis based on specific combinations of motor abnormalities. Regression analyses were used to examine the relationship between kinematic features and clinical variables. Finally, we evaluated the effect of dopaminergic therapy on the ‘bradykinesia complex’ by testing PD individuals both OFF and ON medication.

## 1 Methods

### 1.1 Participants

We enrolled 350 subjects, including 192 participants with PD (age range 40–85 years), and 158 age‐ and sex‐matched HCs (Table 1), at the Department of Human Neurosciences, Sapienza, University of Rome, Italy, and IRCCS Neuromed, Pozzilli (IS), Italy, between March 2015 and March 2024. None of the HCs had a history of neurological or psychiatric disorders, nor were they taking any medications affecting the nervous system. All patients underwent one experimental session after overnight withdrawal of their usual therapy in the case of levodopa, or 36 h or longer in the case of longer‐acting agents (OFF medication). A subset of 129 participants with PD was also tested during their ON medication state. HCs underwent one experimental session. All participants underwent a complete neurological examination, as well as an evaluation with the Montreal Cognitive Assessment (MoCA)^37^ and the Beck's Depression Inventory (BDI) scale.^38^ Motor function in PD individuals was assessed using the Movement Disorder Society‐sponsored revision of the Unified Parkinson's Disease Rating Scale‐Part III (MDS‐UPDRS‐III).^35^, ^36^ Moreover, during the clinical examination, we checked that the participants did not have dystonic hand postures. Levodopa equivalent daily dose (LEDD) was calculated in PD patients.^39^ All participants provided informed consent. The study was approved by the local ethics board and conducted in accordance with the Declaration of Helsinki and international safety guidelines.

**TABLE 1:** Clinical and kinematic data of finger‐tapping in participants with Parkinson's disease (PD), tested with and without their usual dopaminergic therapy (ON and OFF medication), and healthy controls (HCs).

Parameter	HCs (N=158)	PD OFF (N=192)	PD ON (N=129)	*P‐*value*	*P‐*value**
Clinical data					
Sex	56 F	53 F	34 F	0.13	–
Age (years)	66.02 ± 12.6	66.8 ± 9.16	66.2 ± 9.10	0.88	–
MoCA	26.61 ± 4.74	25.59 ± 3.01	27.30 ± 3.14	0.07	–
BDI	5.75 ± 4.97	7.01 ± 5.58	7.14 ± 5.84	**0.04**	–
Disease duration (years)	–	5.27 ± 6.2	4.53 ± 3.6	‐	–
HY stage	–	1.96 ± 0.66	–	–	–
Most affected body side	–	100 R	69 R	–	–
MDS‐UPDRS‐III	–	29.31 ± 12.7	24.45 ± 12.36	–	**<0.001**
LEDD	–	399.64 ± 272.35	407.67 ± 289.32	–	–
Kinematic data					
n° mov	46.26 (16.41)	48.65 (13.89)	46.94 (15.13)	0.15	0.88
Movement rhythm (CV)	0.09 (0.03)	0.14 (0.07)	0.14 (0.07)	**<0.001**	0.40
Movement velocity	1080.80 (252.8)	775.13 (283.26)	874.11 (288.26)	**<0.001**	**<0.001**
Movement amplitude	49.93 (13.48)	40.41 (13.11)	44.01 (12.41)	**<0.001**	**0.001**
Velocity slope	−6.39 (4.73)	−5.48 (4.28)	−5.68 (5.16)	0.06	0.87
Amplitude slope	−0.13 (0.21)	−0.26 (0.31)	−0.27 (0.27)	**<0.001**	0.91

*Note*: Movement velocity is expressed in degrees/second and movement amplitude in degrees. Velocity and amplitude slopes are expressed in (degrees/second)/n° mov and degree/n° mov, respectively. Data are shown as mean values ±1 standard deviation (SD). *P*‐values*, PD OFF condition vs. HCs. *P*‐values**, PD OFF vs. ON medication state. Significant values are shown in bold type.

Abbreviations: HCs, healthy control; PD, Parkinson's disease; F, female; MoCA, Montreal Cognitive Assessment; BDI, Beck Depression Inventory; HY, Hoehn and Yahr stage; R, right; MDS‐UPDRS‐III, Movement Disorder Society‐sponsored revision of the Unified Parkinson's Disease Rating Scale‐Part III; LEDD, levodopa equivalent daily dose; n° mov, number of movements; CV, coefficient of variation.

### 1.2 Kinematic Recording and Analysis

We analyzed the kinematics of repetitive finger‐tapping using a three‐dimensional optoelectronic system (SMART motion system; BTS, Milan, Italy) as detailed elsewhere.^6^, ^7^, ^8^, ^9^, ^10^, ^15^, ^16^, ^20^, ^23^, ^32^, ^40^, ^41^, ^42^, ^43^, ^44^, ^45^, ^46^ Participants were seated comfortably in a chair and instructed to perform repetitive tapping of their index finger on their thumb as widely and as quickly as possible for 15 seconds. Tapping movements were initiated and terminated upon a verbal command. Three consecutive trials of 15 seconds each were recorded. Before the motor task, one practice trial was provided to ensure that participants were familiar with the experimental setting. To prevent fatigue, a rest period of 60 seconds was allowed between trials.^6^, ^7^, ^8^, ^9^, ^10^, ^15^, ^16^, ^20^, ^23^, ^32^, ^40^, ^41^, ^42^, ^43^, ^44^, ^45^, ^46^ Consistent with our previous studies, we recorded finger‐tapping from the most affected hand in PD individuals, and from the dominant hand in HCs; as in our prior research studies, we found no significant effect of handedness on finger‐tapping execution.^6^, ^7^, ^8^, ^9^, ^10^, ^15^, ^16^, ^20^, ^23^, ^32^, ^40^, ^41^, ^42^, ^43^, ^44^, ^45^, ^46^

The collected kinematic data were blindly analyzed using specialized software (SMART Analyzer; BTS). We measured the number of movements (n° mov) within the 15 seconds task window but did not directly measure movement rate to avoid redundancy (since it can be derived from the n° mov/task duration). We also measured the movement rhythm, quantified by the coefficient of variation (CV) of the inter‐tap intervals.^6^, ^7^, ^8^, ^9^, ^10^, ^15^, ^16^, ^20^, ^23^, ^32^, ^40^, ^41^, ^42^, ^43^, ^44^, ^45^, ^46^ We used linear regression techniques to determine the intercept and the slope of the regression line in the scatter plot of kinematic parameters (y‐axis) versus the number of movements (x‐axis).^40^, ^42^, ^43^ Specifically, the intercept represents the movement amplitude (degrees) and velocity (degrees/second) at the beginning of the motor sequence, while the slope describes the average decrement in amplitude, expressed as degree/n° mov and velocity, expressed as (degree/second)/n° mov, that is the sequence effect across the 15 seconds trials.^40^, ^42^, ^43^

### 1.3 Statistical Analysis

#### 1.3.1 Group Comparisons

Statistical comparisons were conducted using the *t*‐test, Mann–Whitney U test, or Fisher's exact test for independent samples, and the paired *t*‐test, Wilcoxon signed‐rank test, or McNemar's test for paired data, as appropriate. The Shapiro–Wilk test assessed data normality. Unless otherwise specified, all results are expressed as mean ± standard deviation (SD). The significance level was set at *P* < 0.05. Data were analyzed using STATISTICA (TIBCO Software Inc., Palo Alto, California, USA) and implemented with R (version 4.4.2).

#### 1.3.2 Clustering Analysis

To further explore data structure, we performed unsupervised clustering on kinematic data collected in the OFF medication state using the mclust package in R (version 4.4.2), which employs Gaussian mixture models for model‐based clustering. The optimal number of clusters was determined using the Bayesian Information Criterion.

#### 1.3.3 ROC Curves Analysis

We used ROC curves to assess the predictive power of each kinematic variable for a diagnostic category (PD OFF vs. HCs), with area under the curve (AUC) indicating discriminative ability. To determine the optimal cut‐off point, we employed the ‘Closest‐to‐(0,1)’ method. Kinematic variables were then dichotomized based on these cut‐offs to classify motor abnormalities, including movement slowness (bradykinesia), reduced movement amplitude (hypokinesia), altered movement rhythm as indicated by higher CV values (dysrhythmia), and amplitude and/or velocity decrement across repetitive movements (sequence effect). Since motor abnormalities could occur independently or in combination, we identified all possible categories, including normal movement, which was defined as any movement for which all kinematic variables fell within the cut‐offs established by ROC curve analysis. We then counted the number of participants in each of the 16 categories across groups and medication states. We compared the distribution of subjects with normal versus abnormal movement using the Fisher's exact and the McNemar test. We used the Mann–Whitney U test and the Wilcoxon signed‐rank test to evaluate differences in the number of bradykinesia features (ranging from 0: normal movement, to 4: all features present) across groups and medication states.

#### 1.3.4 Bayesian Classification

We applied Bayes' theorem to estimate the conditional probability of a participant belonging to the PD (OFF medication) or HC group based on bradykinesia features combinations:

$$P\left(\text{Group}|\text{Feat Comb}\right)=\frac{P\left(\text{Feat Comb}|\text{Group}\right)*P\left(\text{Group}\right)}{P\left(\text{Feat Comb}\right)}$$

Where $P\left(\text{Group}|\text{Feat Comb}\right)$ is the probability that the individual belongs to a certain group (PD or HC), given the specific bradykinesia features combination; $P\left(\text{Feat Comb}|\text{Group}\right)$ is the likelihood of observing a specific combination of features within a particular group, based on the study results; $P\left(\text{Group}\right)$ is the prior probability of an individual being from each group, with $P\left(\mathrm{PD}\right)=0.55$, $P\left(\mathrm{HC}\right)=0.45;\;P\left(\text{Feat Comb}\right)$ is the total probability of seeing a certain bradykinesia features combination across groups.

The same Bayesian approach was used to classify medication states in patients with:

$$P\left(\mathrm{Med}\;\text{State}|\text{Feat Comb}\right)=\frac{P\left(\text{Feat Comb}|\mathrm{Med}\;\text{State}\right)*P\left(\mathrm{Med}\;\text{State}\right)}{P\text{(Feat Comb)}}$$

$\text{with}\;P\left(\mathrm{Med}\;\text{State}\right)\;=\;0.5.$

#### 1.3.5 Regression Analyses

We conducted multiple linear regression to assess the predictive effect of clinical and demographic variables (sex, age, MoCA, BDI, for both groups; additionally, disease duration, LEDD, and MDS‐UPDRS‐III OFF for the PD group) on the dependent variable (number of movement abnormalities). In the ON medication state subsample, we added the independent variable delta MDS‐UPDRS‐III (MDS‐UPDRS‐III OFF minus ON). Stepwise selection models were used with inclusion *F* = 0.05 and removal *F* = 0.1 criteria.

## 2 Data Sharing

The study data are available from the corresponding author upon reasonable request.

## 3 Results

### 3.1 Group Comparisons

**FIG. 1:**
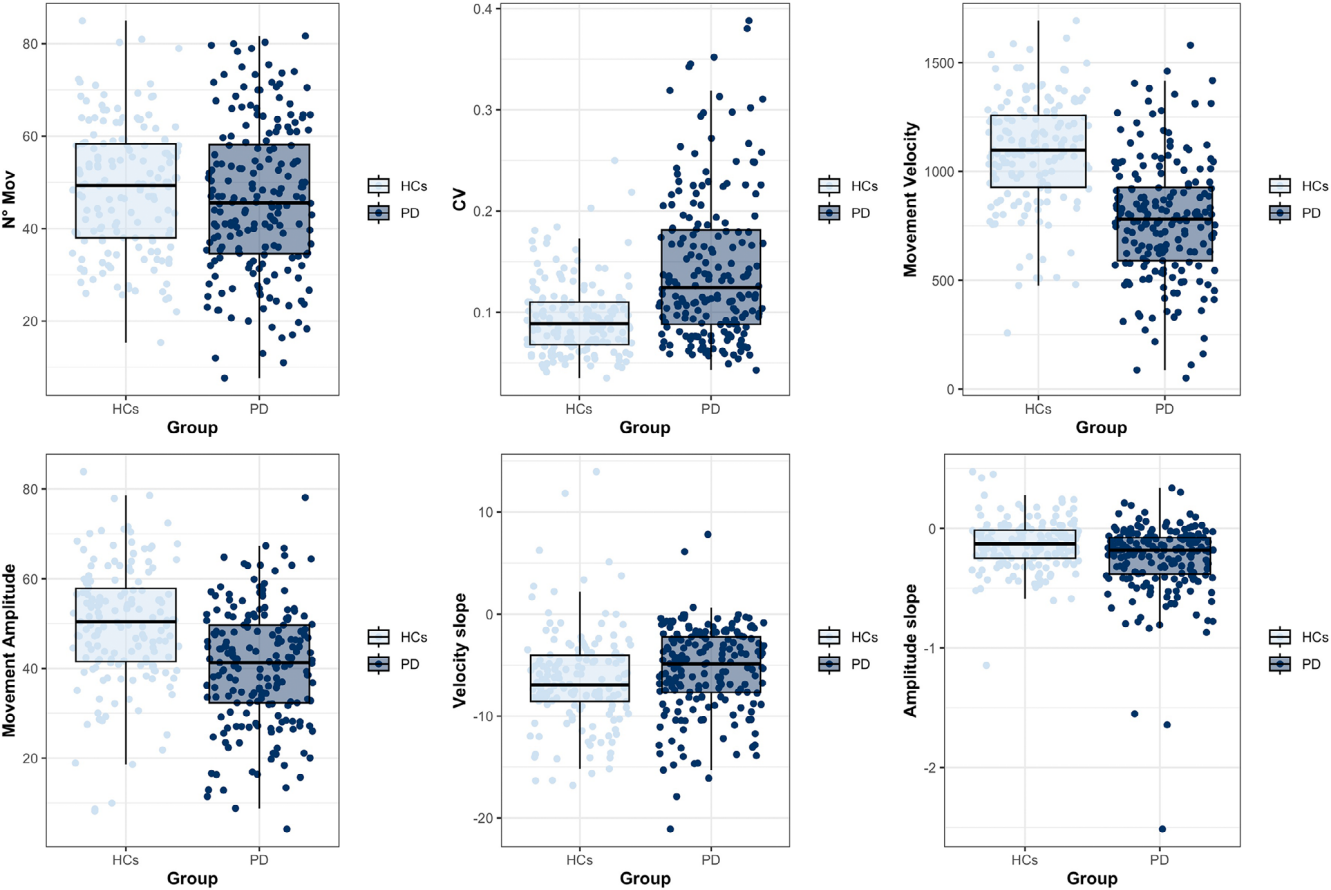
Finger‐tapping kinematics. Although patients with Parkinson's disease (PD) (OFF medication) exhibited lower movement velocity and amplitude, and higher coefficient of variation (CV) and amplitude slope values compared with healthy controls (HCs), a substantial variability and overlap in kinematic measures was observed between the two groups. Movement velocity is expressed in degrees/second, movement amplitude in degrees. Velocity and amplitude slopes are expressed in (degrees/second)/n° mov and degree/n° mov, respectively. n° mov, number of movements. [Color figure can be viewed at wileyonlinelibrary.com]

PD individuals OFF medication had lower movement velocity and amplitude, and higher CV values compared with HCs. We also found differences in terms of amplitude slope, with higher values in PD indicating a greater sequence effect (all *P‐*values < 0.001; Table 1). The number of movements and the velocity slope values (which were not considered in further analysis) did not differ between groups. Consistent with clinical observations^35^, ^36^ and research findings,^3^ these results indicate that velocity decrement is more variable and less consistent than other measures (eg, amplitude decrement), even when assessed objectively, likely due to greater influence from motivational or pharmacological factors.^47^, ^48^ Overall, the raw data inspection demonstrated a significant variability and overlap in the kinematic values between groups (Fig. 1). Comparing PD OFF versus ON states, movement velocity and amplitude were higher ON medication (*P‐*values < 0.001; Table 1), while other parameters showed no changes. As a further analysis, we compared kinematic values in patients stratified according to their Hoehn and Yahr (HY) scores, as in Bologna et al.^40^ Patients in the early stages (HY 1–2) showed higher velocity and amplitude values compared with those in the moderate stages (HY 3–4) (velocity: 808.81 ± 279.30 degrees/second vs. 658.99 ± 242.66 degrees/second; amplitude: 41.58 ± 13.34 degrees vs. 36.24 ± 11.32 degrees; both *P‐*values = 0.01 by Mann–Whitney U test). Patients in the early stages (HY 1–2) also exhibited lower CV values (0.13 ± 0.07 vs. 0.16 ± 0.07, *P* = 0.03). These findings were consistent with lower MDS‐UPDRS‐III scores in the former compared with the latter subgroup (27.12 ± 11.88 vs. 37.89 ± 12.11, *P* < 0.001). No other kinematic parameters differed significantly between subgroups.

### 3.2 Cluster Analysis

**FIG. 2:**
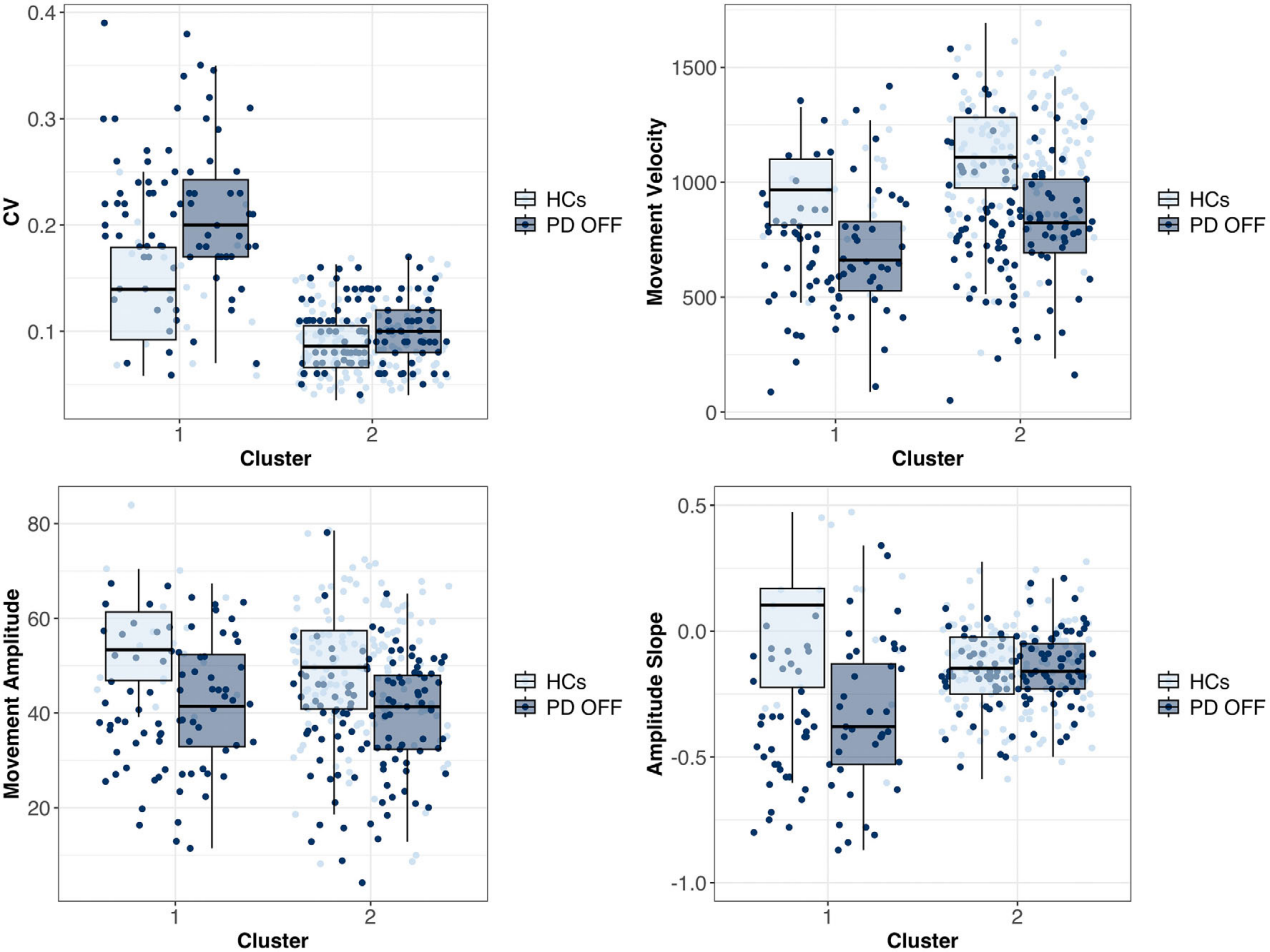
Cluster analysis results. Unsupervised clustering was performed using the mclust package in R (version 4.4.2), which applies Gaussian mixture models for model‐based clustering. The optimal number of clusters was selected using the Bayesian Information Criterion. We identified two clusters as the optimal solution. Cluster 1 was predominantly composed of Parkinson's disease (PD) patients, evaluated OFF medication, with 80 of 98 members belonging to this group. In contrast, Cluster 2 displayed a more balanced distribution, comprising 140 healthy controls (HCs) and 112 PD patients. Movement velocity is expressed in degrees/second, movement amplitude in degrees, amplitude slopes in degree/n° mov. CV, coefficient of variation. [Color figure can be viewed at wileyonlinelibrary.com]

The analysis identified two clusters as the optimal solution (Fig. 2, Table S1). Cluster 1 was predominantly composed of PD individuals, with 80 of 98 members belonging to this group. In contrast, Cluster 2 displayed a more balanced distribution, comprising 140 HCs and 112 PD. The result confirms the visual impression of substantial overlap between patient and control data distributions, with approximately 70% of all subjects grouped in Cluster 2. Comparisons of kinematic parameters between patients in Cluster 1, Cluster 2, and HCs are reported in Table S2.

### 3.3 ROC Curve Analysis

The diagnostic accuracy of kinematic parameters, as indicated by AUC values, was moderate, except for number of movements and velocity slope. Movement velocity was the best discriminator between groups, with 0.77 sensitivity and 0.73 specificity (Supplementary Fig. S1, Table S3). Using ROC‐derived cut‐offs, nearly all PD participants in their OFF medication state (186/192, 96.9%) had at least one movement abnormality. Bradykinesia was the most common feature in PD (148 patients, 77.7%), either alone or in combination with other features, followed by hypokinesia (130 patients, 68.5%) and sequence effect (109 patients, 61.4%), while dysrhythmia was less frequent (59.2%).

**FIG. 3:**
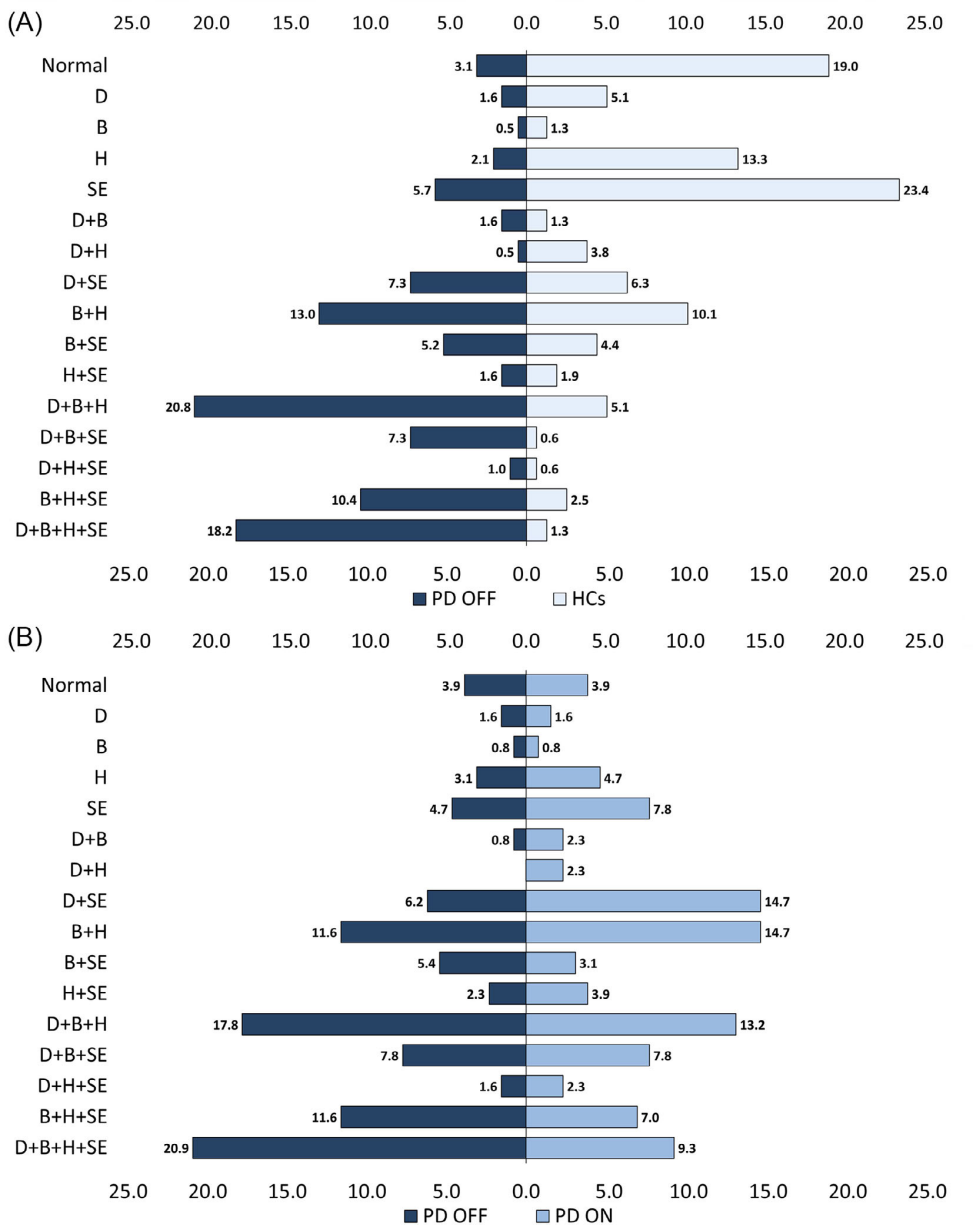
Bradykinesia features combinations in individuals with Parkinson's disease (PD) and healthy controls (HCs). Numbers indicate percentages. The motor alterations were determined in each study participant based on the cut‐offs from the analysis of the receiver operating characteristic (ROC) curves. (A) Data from 192 PD patients tested in the OFF medication (ie, without their usual dopaminergic therapy) (left) and 158 HCs (right). (B) Data collected in a subsample of 129 PD patients tested with (ON, right) and without (OFF, left) their usual dopaminergic therapy. D, dysrhythmia; B, bradykinesia (movement slowness); H, hypokinesia (low amplitude movement); SE, sequence effect (amplitude slope). [Color figure can be viewed at wileyonlinelibrary.com]

**FIG. 4:**
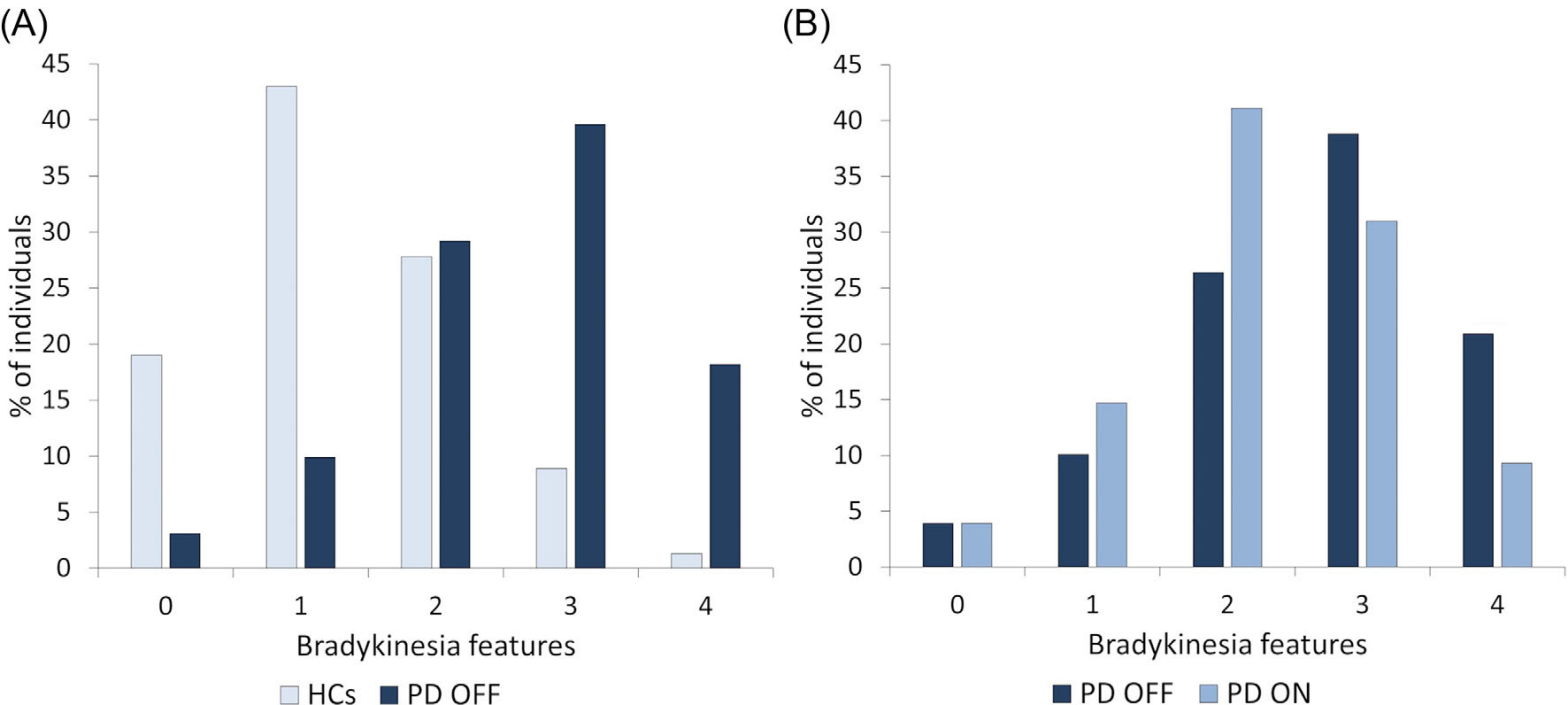
Number of bradykinesia features in Parkinson's disease (PD) individuals and elderly healthy controls (HCs). (A) Data from 192 PD patients tested without their usual dopaminergic therapy (OFF condition) and 158 HCs. (B) Data collected in a subsample of 129 PD patients tested with and without their usual dopaminergic therapy (ON and OFF condition). NB. The number of bradykinesia features differed between patients and controls (*P* < 0.001 by Mann–Whitney U test), and dopaminergic therapy impacted on the number of bradykinesia features in patients (*P* < 0.001 by Wilcoxon signed‐rank test). [Color figure can be viewed at wileyonlinelibrary.com]

Most PD individuals exhibited combinations of motor abnormalities. Bradykinesia frequently co‐occurred with hypokinesia, while sequence effect co‐occurred with dysrhythmia. The co‐occurrence of all four motor abnormalities was observed in nearly 20% of PD patients (Figs 3A, 4A, Table S4). Upon examining specific combinations with particular reference to current PD criteria,^1^ the combination of bradykinesia and sequence effect, alongside additional motor abnormalities, was present in only 79 of 192 patients (41%), and only 5.2% exhibited bradykinesia and sequence effect (Fig. 3A, Table S4).

Although lower than in PD (*P* < 0.001), a notable proportion of HCs (38/158, 81%) also exhibited finger‐tapping abnormalities. The most common features in HCs were the sequence effect (65 individuals, 34%), followed by hypokinesia (61 individuals, 31.9%), and bradykinesia (42 individuals, 21.7%). Dysrhythmia was observed in 32 HCs (16.7%). The number of motor abnormalities differed between groups (*P* < 0.001). Unlike PD, most HCs exhibited only one (43%) or two (27.8%) abnormalities (Figs 3A, 4A, Table S4). When combinations occurred, the most common were bradykinesia and hypokinesia, or sequence effect plus dysrhythmia. Only 8.9% of HCs exhibited three motor abnormalities, while only 1.3% had all four features.

The total number of patients with bradykinesia (99/129 OFF vs. 75/129 ON, *P* < 0.001) and hypokinesia (89/129 OFF vs. 75/129 ON, *P* < 0.001), alone or combined, decreased with therapy. The number of patients showing the sequence effects was slightly modified by therapy (78 OFF vs. 73 ON, *P* = 0.03), while the number of patients with dysrhythmia remained unchanged (73/129 OFF vs. 67/129 ON, *P* = 0.17). Although the overall number of patients with abnormal movement did not change (124 OFF vs. 124 ON, *P* = 1), dopaminergic therapy significantly impacted on the number of bradykinesia features (*P* < 0.001 by Wilcoxon signed‐rank test) (Figs 3B and 4B). However, treatment did not alter the specific motor features combinations (Fig. 3B, Table S5).

### 3.4 Bayesian Classification

The most distinctive motor pattern for PD diagnosis was the combination of all four motor features, which yielded a 94.6% probability of PD and only a 5.4% probability of HC (Table S6, Fig. S2A). The second most predictive combination consisted of bradykinesia, sequence effect, and dysrhythmia (93.3% probability of PD). Other three‐feature combinations, such as bradykinesia, sequence effect, and hypokinesia, or bradykinesia, hypokinesia, and dysrhythmia, both had an 83.3% probability of PD. Conversely, the combination of bradykinesia and sequence effect, as for the current clinical criteria for PD, had a 78.1% probability of PD. Isolated abnormalities (eg, sequence effect or hypokinesia alone) were more indicative of HC (Table S6, Fig. S2A). Bayes' theorem also revealed that the presence of all four motor features indicated the OFF state (69.2% probability). Conversely, the presence of two abnormalities was more characteristic of the ON state (Table S7, Fig. S2B).

Bayesian results in the two clusters are detailed in Tables S8 and S9. In Cluster 2, which included more than 70% of cases, the combination of bradykinesia and sequence effect^1^ without additional motor abnormalities had no discriminatory power between PD and HCs (Table S9).

### 3.5 Regression Analysis

The analysis revealed a significant model explaining 14.8% of the variance in the number of motor abnormalities in the whole PD sample. The MDS‐UPDRS‐III score was a moderate‐to‐strong positive predictor of motor impairment, demonstrating a large effect size (ie, clinical motor scores predict the number of bradykinesia features). In contrast, MoCA scores and age were negative predictors (small‐to‐moderate effect size; Table S10). In the ON medication state subgroup, LEDD was another negative predictor of motor impairment, with moderate‐to‐strong effect size (ie, lower LEDD was associated with a higher number of bradykinesia features) (Tables S10, S11). No regression model was established in the HCs group, indicating that none of the independent variables predicted the number of bradykinesia features.

## 4 Discussion

Our study represents the most comprehensive kinematic investigation of bradykinesia and related features (ie, the ‘bradykinesia complex’) in PD participants and elderly controls conducted to date. Namely, bradykinesia, defined as slowness of movement, emerged as the most prevalent motor abnormality in patients and the most accurate feature for distinguishing them from HCs. Notably, bradykinesia discriminatory power increased significantly when combined with other motor features (ie, hypokinesia, dysrhythmia, and sequence effect). In contrast to PD, HCs typically exhibited isolated motor alterations. Lastly, dopaminergic treatment had differential effects on bradykinesia and its associated features in patients. Our results highlight that identifying multiple movement abnormalities is crucial for accurately differentiating PD individuals from healthy individuals, underscoring the importance of evaluating all motor features separately for diagnostic purposes. The findings have clinical and pathophysiological implications.

### 4.1 Insights into the ‘Bradykinesia Complex’

The key study finding is the objective demonstration that, despite a significant overlap between patient and control data, most PD participants exhibited multiple concurrent motor abnormalities, whereas most HCs had isolated abnormalities. Using a Bayesian approach, we demonstrated that the combination of bradykinesia, hypokinesia, dysrhythmia, and sequence effect was associated with a 94.6% a priori probability of PD, compared with only a 5.4% probability of being an HC. Notably, other frequent combinations of three motor abnormalities in PD included: (i) bradykinesia, hypokinesia, and dysrhythmia; (ii) bradykinesia, dysrhythmia, and sequence effect; and (iii) bradykinesia, hypokinesia, and sequence effect, each associated with an a priori PD probability ranging from 83% to 93%. Less commonly, PD patients presented with only two motor abnormalities. In these cases, two patterns emerged: (i) bradykinesia combined with hypokinesia and (ii) sequence effect combined with dysrhythmia. These findings offer insights into the concept of the ‘bradykinesia complex’ and may be interpreted by considering both quantitative aspects (ie, the number of motor abnormalities) and qualitative ones, that is, the specific combinations observed.

Regarding the significance of the number of motor abnormalities in combinations observed in PD individuals, our regression analysis showed that a higher number of abnormalities was associated with higher clinical motor scores. This suggests that individuals with a more complex combination of bradykinesia features exhibit a more severe clinical picture. Accordingly, we observed that the number of bradykinesia features predicted lower cognitive scores, according to previous findings.^8^, ^20^, ^41^ Interestingly, the number of bradykinesia features in individual patients did not correlate with disease duration, indicating that the extent of impairment within the ‘bradykinesia complex’ is not merely a consequence of disease progression. This interpretation is further supported by the lack of correlation between the number of bradykinesia features and age. In fact, although with a small‐to‐moderate effect size, age emerged as a negative predictor of the number of abnormalities, meaning that younger patients in our cohort were more likely to present with a greater number of bradykinesia features. Overall, these findings allow the hypothesis that PD subtypes may be independent of disease duration and may be driven by different underlying pathophysiological mechanisms.

Concerning the specific bradykinesia features combinations, the observation of bradykinesia and sequence effect, as specified by the MDS consensus criteria,^1^ was relatively low among PD subjects, especially when compared with other types of combinations. Again, the sequence effect was absent in a substantial proportion of patients. These observations confirm previous findings from smaller cohorts^3^, ^6^, ^40^ and call into question the definition of bradykinesia (movement slowness and the sequence effect) in the context of current clinical criteria for PD.^1^ In this regard, using ‘bradykinesia and associated features’^2^ appears more appropriate as it better reflects the heterogeneous spectrum of motor abnormalities observed in PD. Furthermore, this proposed definition could also be applied when considering patients with very advanced PD or in the context of atypical parkinsonism, such as progressive supranuclear palsy, where the sequence effect is generally absent, most likely due to hypokinesia.^3^, ^49^, ^50^ Regarding observations in the elderly population, it is important to recognize that bradykinesia and associated features can also be present in healthy aging individuals, albeit often in a different form and pattern. We observed that a substantial number of HCs typically exhibit isolated motor abnormalities,^15^, ^51^, ^52^, ^53^ raising considerations regarding the variability of motor function in aging populations and the overlap with PD. Previous studies demonstrated that mild parkinsonian signs (MPS), including subtle bradykinesia features, may occur in up to 30–40% of otherwise healthy elderly individuals.^1^, ^6^, ^13^, ^14^, ^15^ Although their clinical significance remains unclear, the presence of MPS in older adults has been interpreted as a potential early indicator of underlying neurodegenerative changes.^13^, ^14^, ^51^, ^54^, ^55^, ^56^ Longitudinal studies have linked MPS to a higher risk of developing PD, cognitive decline, and other neurodegenerative diseases.^5^, ^51^, ^52^, ^57^, ^58^, ^59^ Neuroimaging has confirmed structural and functional alterations in motor‐related regions and dopaminergic pathways in individuals with MPS.^5^, ^52^, ^53^, ^59^, ^60^ Beyond the dopaminergic system, other neural circuits also play a role in aging motor disturbances, including the cholinergic system, which is essential for motor coordination, cognitive function, and overall neural regulation. Acetylcholine modulates both motor cortex and the basal ganglia activity, interacting with cortico‐striatal circuits to influence dopaminergic transmission and, consequently, movement execution.^7^, ^8^, ^15^, ^61^ Neuroimaging studies have shown a reduction in cholinergic innervation, particularly in the basal nucleus of Meynert, which correlates with both motor and cognitive deficits.^62^ Supporting the role of the cholinergic system in the pathophysiology of bradykinesia, our previous studies,^7^, ^8^ consistent with other research groups,^63^, ^64^, ^65^ showed that bradykinesia and dysrhythmia also occur in patients with Alzheimer's disease and mild cognitive impairment. We also found a correlation between movement velocity and neurophysiological measures of cholinergic activity (short‐latency afferent inhibition). However, apart from some reports on gait step‐time variability in PD,^66^ no published studies have specifically examined how the cholinergic system modulates the bradykinesia features.

An additional intriguing finding is the high prevalence of an isolated sequence effect in healthy elderly individuals. Hence, isolated sequence effect may represent a form of MPS associated with aging.^40^ The findings suggest that the sequence effect may be a nonspecific phenomenon, also present to some extent in healthy elderly individuals. This questions its diagnostic specificity and implies it could be part of broader age‐related motor changes. For example, the sequence effect in HCs may partly reflect a fatigue effect associated with aging. Further investigations, however, are needed to better characterize MPS in aging people.

### 4.2 Pathophysiological Implications

From the pathophysiological standpoint, our results support the hypothesis of bradykinesia as a network disorder.^2^, ^3^, ^4^ In this framework, bradykinesia and each associated feature may reflect the differential involvement of specific nodes within this network, which includes the basal ganglia, the sensorimotor cortical areas, and the cerebellum.^2^, ^3^, ^4^ Moreover, it can be hypothesized that certain alterations tend to co‐occur preferentially in patients because they are linked to similar pathophysiological mechanisms (eg, bradykinesia and hypokinesia arise from mechanisms distinct from those underlying the sequence effect and dysrhythmia).^40^, ^42^, ^47^, ^67^, ^68^, ^69^, ^70^, ^71^

Bradykinesia and hypokinesia share some common mechanisms related to abnormal basal ganglia activity (eg, altered β‐band oscillations within basal ganglia–cortical loops), along with other (possibly compensatory) changes at the cortical or cerebellar level. Evidence indicates that basal ganglia–cortical loops specifically regulate movement velocity and amplitude, with dopamine providing the necessary drive.^3^ When dopamine levels fall below a critical threshold, bradykinesia and hypokinesia emerge, with enhanced β‐band oscillations representing a hallmark of this dysfunction.^43^, ^72^, ^73^, ^74^, ^75^ Consequently, dopaminergic therapy and surgical interventions modulating basal ganglia–cortical loops’ oscillatory activity effectively improve these motor features. Furthermore, modulation of beta oscillatory activity through non‐invasive stimulation techniques has also been shown to significantly impact bradykinesia in individuals with PD^43^. Alternatively, the co‐presence of bradykinesia and hypokinesia might be explained by biomechanical principles, given that movement velocity is typically proportional to movement amplitude under normal conditions, a relationship that can be altered in PD.^43^, ^76^ Rather than reflecting basal ganglia abnormalities, dysrhythmia and the sequence effect are thought to primarily arise from abnormal activity in cortical and cerebellar circuits, which play a key role in movement timing and feedback, especially in sustaining repetitive and continuous actions.^3^ Accordingly, neurophysiological and neuroimaging studies have highlighted pathophysiological differences between PD participants with and without the sequence effect, particularly in cortical and cerebellar regions.^2^, ^3^, ^68^, ^77^ Dysrhythmia and the sequence effect may therefore emerge when compensatory mechanisms within cortical and cerebellar circuits fail.^3^, ^4^ As a result, therapeutic improvement with dopaminergic or surgical interventions for dysrhythmia and the sequence effect is likely to be limited.^23^, ^25^, ^42^, ^43^, ^69^, ^71^

Hence, bradykinesia, hypokinesia, dysrhythmia, and the sequence effect exhibit variable sensitivity to dopaminergic replacement and to surgical interventions, likely due to the multilevel network effects of such treatments.^3^, ^42^, ^78^ Moreover, the different characteristics of bradykinesia respond differently to various types of stimulation, such as high‐ or low‐frequency deep brain stimulation.^31^

Finally, from the pathophysiological perspective, the finding of age as a negative predictor of the number of abnormalities in patients (ie, lower age is associated with more abnormalities) can be interpreted in the light of age‐related changes in brain connectivity, as aging is associated with reduced network segregation and flexibility.^79^ One possible explanation is that in younger patients, relatively preserved and responsive brain networks may engage more actively when affected by pathology, resulting in a broader combination of motor features. Nevertheless, this finding should be interpreted with caution, as it may reflect an epiphenomenon due to unknown confounds. Clarifying this issue warrants further investigation.

### 4.3 Confounds and Study Limitations

Given the demographic similarities between the PD and HCs, we exclude these factors as confounders. PD diagnosis was based on clinical criteria, and although not all patients underwent dopamine transporter (DAT) single‐photon emission computed tomography (SPECT), long‐term monitoring minimized misdiagnosis bias.^1^, ^80^ Moreover, we did not perform DAT SPECT in HCs, as there was no clinical indication. To assess bradykinesia, we utilized finger‐tapping, the most valuable task in clinical practice.^2^, ^3^, ^4^, ^35^, ^36^, ^42^ Notably, we have used an approach thoroughly validated in several prior studies.^6^, ^8^, ^9^, ^10^, ^16^, ^32^, ^40^, ^41^, ^42^, ^44^, ^45^, ^81^ However, we did not investigate bradykinesia features during other tasks,^3^, ^4^ including more complex tasks such as sequential finger‐tapping^82^, ^83^ or writing (eg, to detect progressive or consistent micrographia^3^, ^68^), nor did we examine possible alterations in other body regions,^68^, ^84^, ^85^ or in spontaneous (non‐task related) movements,^3^, ^84^, ^86^ (ie, oligokinesia).^2^ Future studies should explore the clinical relevance of these alternative measures. We tested PD participants both ON and OFF medication to minimize the potential confounding effects of drugs on the kinematic variables. Finally, we ensured that the researchers who performed kinematic analyses were blinded to the participants' diagnosis and experimental condition. Despite the relatively large sample, some of the 16 predefined categories included few subjects, which may make the Bayesian analysis results less reliable. The cluster analysis was carried out to demonstrate the variability and overlap within the dataset, rather than to pursue in‐depth analyses of patient subgroups. Future studies will address the bradykinesia complex in more homogeneous patient subgroups. Additional limitations include the focus on intermediate‐phase PD, which restricts the generalizability of the results to very advanced disease stages, and the absence of a longitudinal design. Finally, although our cohort included 24 individuals with early‐onset PD, genetic abnormalities were not assessed. This represents another study limitation and an area of future research.

## 5 Conclusions

This study provides new insights into the ‘bradykinesia complex’ and its challenges both in clinical and research settings. For example, the finding that a considerable proportion of PD individuals displayed only one or two motor abnormalities, with substantial overlap with healthy subjects, highlights a critical limitation of the current clinical approach to bradykinesia evaluation, underscoring the need for refined assessment methods. In this regard, assessing the clinical relevance of multiple movement abnormalities, regardless of their specific combination, may represent a novel approach for defining parkinsonism and PD diagnosis. Our findings also show that different motor abnormalities vary in treatment response, supporting the view that bradykinesia and associated features arise from dysfunction across multiple brain regions. Clinically, this underscores PD heterogeneity and suggests that detailed motor phenotyping may have therapeutic implications, as some motor abnormalities respond differently to dopaminergic therapy. Future research, possibly complemented by neurophysiological and neuroimaging methods, is essential to further investigate the ‘bradykinesia complex.’

## Author Roles

(1) Research Project: A. Conceptualization, B. Organization, C. Data Curation; (2) Statistical Analysis: A. Design, B. Formal Analysis, C. Review and Critique; (3) Manuscript Preparation: A. Writing of the First Draft, B. Review and Critique; (4). A. Supervision.

G.P.: 1A, 1C, 2B, 3A.

M.D.R.: 1C, 2B, 3B.

A.C.: 1C, 3B.

D.B.: 1C, 3B.

D.C.: 1C, 3B.

L.A.: 1C, 2B, 3B.

D.A.F.: 2B, 3B.

A.F.: 2C, 3B

A.J.E.: 2C, 3B.

M.B.: 1A, 1B, 1C, 2A, 2C, 3B, 4A.

## Financial Disclosures of All Authors (for the Preceding 12 months)

A.F. has stock ownership in Inbrain Pharma and has received payments as consultant and/or speaker from AbbVie, Abbott, AskBio, Boston Scientific, Ceregate, Dompé Farmaceutici, Inbrain Neuroelectronics, Ipsen, Medtronic, Iota, Syneos Health, Merz, Sunovion, Paladin Labs, UCB, and Sunovion. He has received research support from AbbVie, Boston Scientific, Medtronic, Praxis, and ES; and receives royalties from Springer. A.J.E. has received grant support from the National Institutes of Health (NIH) and The Michael J. Fox Foundation for Parkinson's Research; personal compensation as a consultant/scientific advisory board member for Mitsubishi Tanabe Pharma America (formerly Neuroderm), Amneal, Acorda, AbbVie, Bial, Supernus (formerly USWorldMeds), NeuroDiagnostics, Inc. (SYNAPS Dx), Intrance Medical Systems, Inc., Merz, Praxis Precision Medicines, Citrus Health, and Herantis Pharma; Data Safety Monitoring Board (Chair) of AskBio; and publishing royalties from Lippincott Williams & Wilkins, Cambridge University Press, and Springer. He is co‐inventor of the patent “Compositions and methods for treatment and/or prophylaxis of proteinopathies.” He cofounded REGAIN Therapeutics to fund preclinical studies but relinquished the right to any personal income from future treatments. He serves on the editorial boards of *Journal of Parkinson's Disease, Journal of Alzheimer's Disease*, *European Journal of Neurology, Movement Disorders Clinical Practice*, and *JAMA Neurology*.

## Supporting information


**Table S1.** Cluster analysis. Unsupervised clustering was performed using the mclust package in R (version 4.4.2), which applies Gaussian mixture models for model‐based clustering. The optimal number of clusters was selected using the Bayesian Information Criterion. We identified two clusters as the optimal solution. Cluster 1 was predominantly composed of Parkinson's disease (PD) patients, with 80 of 98 members belonging to this group. In contrast, Cluster 2 displayed a more balanced distribution, comprising 140 healthy controls (HCs) and 112 PD patients. The total number of subjects is indicated in square brackets. Min, minimum value; Q1, first quartile, the value below which 25% of the data fall; Me, median; Q3, third quartile; Max, maximum value; CV, coefficient of variation. Movement velocity is expressed in degrees/second, movement amplitude in degrees, amplitude slope in degree/n° mov. n° mov, number of movements.
**Table S2:** Comparisons of kinematic parameters between Parkinson's disease (PD) patients belonging to Cluster 1, Cluster 2, and healthy controls (HCs). Note that the one‐way analyses of variance (ANOVAs) revealed significant group effects across all kinematic variables. Post hoc comparisons revealed that for the number of movements, HCs differed from Cluster 1 but not Cluster 2, while Cluster 1 showed lower values than Cluster 2. Regarding the coefficient of variation (CV), HCs differed from Cluster 1 but not Cluster 2, with Cluster 1 exhibiting higher rhythm variability. Movement velocity was reduced in both clusters compared with HCs, with Cluster 1 showing lower values than Cluster 2. In contrast, movement amplitude differed between HCs and both clusters, but no significant difference was found between Clusters 1 and 2. For the velocity slope, HCs differed from Cluster 2 but not Cluster 1, although Cluster 1 showed a greater velocity decrement than Cluster 2. Similarly, in the amplitude slope, HCs differed from Cluster 1 but not Cluster 2, with Cluster 1 again showing a greater amplitude decrement. n° mov, number of movements; P, HCs vs. Cluster 1 PD; P**, HCs vs. Cluster 2 PD; P***, Cluster 1 vs. Cluster 2 PD.
**Table S3:** Kinematic cut‐off values obtained from the receiver operating characteristics (ROC) curves analysis. Movement velocity is expressed in degrees/second, movement amplitude in degrees, velocity slope in (degree/second)/n mov, amplitude slope in degree/n mov. n° mov, number of movements; CV, coefficient of variation; AUC, area under the curve.
**Table S4:** Bradykinesia features in patients with Parkinson's disease (PD) and healthy controls (HCs). The total number of subjects included in the two groups is indicated in square brackets. Percentages are in parentheses. D, dysrhythmia; B, bradykinesia (movement slowness); H, hypokinesia (low amplitude movement); SE, sequence effect (amplitude slope).
**Table S5:** Bradykinesia features in patients with Parkinson's disease (PD) tested after overnight withdrawal (OFF medication) and with their usual dopaminergic therapy (ON medication). The total number of subjects is indicated in square brackets. Percentages are in parentheses. D, dysrhythmia; B, bradykinesia (movement slowness); H, hypokinesia (low amplitude movement); SE, sequence effect (amplitude slope).
**Table S6:** Conditional probabilities of a participant belonging to either Parkinson's disease (PD) or healthy controls (HCs) groups based on specific bradykinesia features, their combinations, and number of features (feat count). D, dysrhythmia; B, bradykinesia (movement slowness); H, hypokinesia (low amplitude movement); SE, sequence effect (amplitude slope).
**Table S7:** Conditional probabilities of a Parkinson's disease (PD) participant belonging to either the OFF and ON medication state based on specific bradykinesia features, their combinations, and number of features (feat count). D, dysrhythmia; B, bradykinesia (movement slowness); H, hypokinesia (low amplitude movement); SE, sequence effect (amplitude slope).
**Table S8:** Bradykinesia features in participants belonging to Cluster 1. The total number of subjects included in the two groups are indicated in square brackets. Percentages are in parentheses. Numbers in italics indicate the conditional probabilities of a participant belonging to either the Parkinson's disease (PD) or healthy controls (HCs) groups based on specific bradykinesia features, their combinations (feat comb), and number of features (feat count). Note that Cluster 1 included more PD patients than HCs. Compared with HCs, a higher number of patients showed abnormal movements (*P* = 0.02 by Fisher's exact test). Finally, the number of bradykinesia features differed between PD and HCs included in Cluster 1 (*P* < 0.001 by Mann–Whitney U test). D, dysrhythmia; B, bradykinesia (movement slowness); H, hypokinesia (low amplitude movement); SE, sequence effect.
**Table S9:** Bradykinesia features in participants belonging to Cluster 2. The total number of subjects included in the two groups are indicated in square brackets. Percentages are in parentheses. *P*‐values from Mann–Whitney U test. Significant values are shown in bold. Numbers in italics indicate the conditional probabilities of a participant belonging to either the Parkinson's disease (PD) or healthy controls (HCs) groups based on specific bradykinesia features, their combinations (feat comb), and number of features (feat count). In Cluster 2, we noted a roughly equal representation from the PD and HCs. Compared with HCs, a higher number of patients showed abnormal movements (*P* = 0.005 by Fisher's exact test). The number of bradykinesia features differed between PD and HCs included in Cluster 2 (*P* < 0.001 by Mann–Whitney U test). Note that in Cluster 2, the combination of bradykinesia and sequence effect without additional features had no discriminatory power. D, dysrhythmia; B, bradykinesia (movement slowness); H, hypokinesia (low amplitude movement); SE, sequence effect (amplitude slope).
**Table S10.** Regression analysis conducted in Parkinson's disease (PD) patients. The analysis revealed a statistically significant model that explained 16.1% of the variance in the number of motor abnormalities (adjusted R^2^ = 0.148, F[3, 188] = 12.06, *P* < 0.001). Other variables, including sex, Beck Depression Inventory (BDI), disease duration, levodopa equivalent daily dose (LEDD), and Hoehn and Yahr stage were excluded from the model as they did not significantly enhance its explanatory power. MDS‐UPDRS‐III, Movement Disorder Society‐sponsored revision of the Unified Parkinson's Disease Rating Scale‐Part III; LEDD, levodopa equivalent daily dose; MoCA, Montreal Cognitive Assessment.
**Table S11.** Regression analysis conducted in the subsample of 129 Parkinson's disease (PD) patients tested in the ON and OFF medication state. The analysis revealed a statistically significant model that explained 24.4% of the variance in the number of motor abnormalities (adjusted R^2^ = 0.244, F[5, 123] = 9.28, *P* < 0.001). Other variables, including disease duration, Beck Depression Inventory (BDI) scores, and delta Movement Disorder Society‐sponsored revision of the Unified Parkinson's Disease Rating Scale‐Part III (MDS‐UPDRS‐III) were excluded from the model as they did not significantly enhance its explanatory power. LEDD, levodopa equivalent daily dose; MoCA, Montreal Cognitive Assessment.
**Figure S1:** Receiver operating characteristic (ROC) curves. ROC curves were used to graphically represent the diagnostic properties of the finger‐tapping kinematic variables (number of movements, ie, n° mov; coefficient of variation, ie, CV; movement velocity and amplitude, velocity, and amplitude slope, ie, sequence effect). The value of the area under the ROC curve (AUC) measures how well the model can discriminate between subjects. To determine the optimal cut‐off point, we employed the ‘Closest‐to‐(0,1)’ method, which minimizes the Euclidean distance between the ROC curve and the ideal point (0,1) on the ROC plane. This method optimally balances sensitivity (true positive rate) and specificity (true negative rate) for classification.
**Figure S2:** Conditional probabilities. We calculated the conditional probabilities of a participant belonging to (A) either the Parkinson's disease (PD) and healthy controls (HCs) groups or (B) the OFF and ON medication state based on specific bradykinesia features and combinations.

## Data Availability

The data that support the findings of this study are available from the corresponding author upon reasonable request.
