# Supplementary material for: Analyzing the ‘Bradykinesia Complex’ in Parkinson's Disease
Source: Mov Disord. 2025 Oct 17;41(1):143–55. doi: 10.1002/mds.70082 (PMC12882039; doi:10.1002/mds.70082)
Supplement: Supplementary file 1 — Table S1. Cluster analysis. Unsupervised clustering was performed using the mclust package in R (version 4.4.2), which applies Gaussian mixture models for model‐based clustering. The optimal number of clusters was selected using the Bayesian Information Criterion. We identified two clusters as the optimal solution. Cluster 1 was predominantly composed of Parkinson's disease (PD) patients, with 80 of 98 members belonging to this group. In contrast, Cluster 2 displayed a more balanced distribution, comprising 140 healthy controls (HCs) and 112 PD patients. The total number of subjects is indicated in square brackets. Min, minimum value; Q1, first quartile, the value below which 25% of the data fall; Me, median; Q3, third quartile; Max, maximum value; CV, coefficient of variation. Movement velocity is expressed in degrees/second, movement amplitude in degrees, amplitude slope in degree/n° mov. n° mov, number of movements. Table S2: Comparisons of kinematic parameters between Parkinson's disease (PD) patients belonging to Cluster 1, Cluster 2, and healthy controls (HCs). Note that the one‐way analyses of variance (ANOVAs) revealed significant group effects across all kinematic variables. Post hoc comparisons revealed that for the number of movements, HCs differed from Cluster 1 but not Cluster 2, while Cluster 1 showed lower values than Cluster 2. Regarding the coefficient of variation (CV), HCs differed from Cluster 1 but not Cluster 2, with Cluster 1 exhibiting higher rhythm variability. Movement velocity was reduced in both clusters compared with HCs, with Cluster 1 showing lower values than Cluster 2. In contrast, movement amplitude differed between HCs and both clusters, but no significant difference was found between Clusters 1 and 2. For the velocity slope, HCs differed from Cluster 2 but not Cluster 1, although Cluster 1 showed a greater velocity decrement than Cluster 2. Similarly, in the amplitude slope, HCs differed from Cluster 1 but not Cluster 2, with Clus [file MDS-41-143-s001.docx]

**SUPPLEMETARY MATERALS**

|  | **Cluster 1 [98]** | | | | | **Cluster 2 [252]** | | | | |
| --- | --- | --- | --- | --- | --- | --- | --- | --- | --- | --- |
|  | CV  CV | | | | | | | | | |
|  | Min | Q1 | Me | Q3 | Max | Min | Q1 | Me | Q3 | Max |
| HCs | 0.058 | 0.092 | 0.140 | 0.179 | 0.250 | 0.035 0.066 0.086 0.105 0.169 | 0.066 0.066 0.086 0.105 0.169 | 0.086 0.066 0.086 0.105 0.169 | 0.105 0.066 0.086 0.105 0.169 | 0.169 0.066 0.086 0.105 0.169 |
| PD | 0.059 | 0.170 | 0.200 | 0.242 | 0.390 | 0.040 | 0.080 | 0.100 | 0.120 | 0.170 |
|  | MOVEMENT VELOCITY  MOVEMENT VELOCITY | | | | | | | | | |
|  | Min | Q1 | Me | Q3 | Max | Min | Q1 | Me | Q3 | Max |
| HCs | 476.0 | 813.6 | 967.1 | 1100.8 | 1327.2 | 257.6 | 974.3 | 1108.2 | 1282.6 | 1693.1 |
| PD | 87.2 | 526.8 | 660.7 | 829.1 | 1418.1 | 50.4 | 692.9 | 823.6 | 1012.9 | 1580.6 |
|  | MOVEMENT AMPLITUDE  MOVEMENT AMPLITUDE | | | | | | | | | |
|  | Min | Q1 | Me | Q3 | Max | Min | Q1 | Me | Q3 | Max |
| HCs | 39.156 | 46.858 | 53.344 | 61.324 | 83.858 | 8.169 | 40.835 | 49.667 | 57.399 | 78.551 |
| PD | 11.430 | 32.912 | 41.400 | 52.320 | 67.361 | 4.170 | 32.348 | 41.315 | 47.967 | 78.100 |
|  | AMPLITUDE SLOPE  AMPLITUDE SLOPE | | | | | | | | | |
|  | Min | Q1 | Me | Q3 | Max | Min | Q1 | Me | Q3 | Max |
| HCs | -1.145 | -0.276 | 0.102 | 0.168 | 0.473 | -0.588 | -0.250 | -0.148 | -0.025 | 0.276 |
| PD | -2.510 | -0.557 | -0.385 | -0.138 | 0.340 | -0.540 | -0.230 | -0.160 | -0.050 | 0.210 |

**Supplementary Table 1: Cluster analysis.** Unsupervised clustering was performed using the mclust package in R (version 4.4.2), which applies Gaussian Mixture Models (GMM) for model-based clustering. The optimal number of clusters was selected using the Bayesian Information Criterion (BIC). We identified two clusters as the optimal solution. Cluster 1 was predominantly composed of Parkinson’s disease patients (PD), with 80 out of 98 members belonging to this group. In contrast, Cluster 2 displayed a more balanced distribution, comprising 140 healthy controls (HCs) and 112 PD. The total number of subjects is indicated in square brackets. Min: Minimum value, Q1: first quartile — the value below which 25% of the data fall (also called the 25th percentile). Me: Median, Q3: third quartile, Max: Maximum value. CV: coefficient of variation. Movement velocity is expressed in degrees/sec, movement amplitude in degrees, amplitude slopes in (degree/n mov).

|  | **ANOVA (F, df)** | **p** | **p**** | **p***** |
| --- | --- | --- | --- | --- |
| N° MOV | F_2, 347_=7.26, P<0.001 | **0.001** | 0.6 | **0.001** |
| CV | F_2, 347_=173.15, P<0.001 | **<0.001** | 0.27 | **<0.001** |
| MOVEMENT VELOCITY | F_2, 347_=65.34, P<0.001 | **<0.001** | **<0.001** | **0.001** |
| MOVEMENT AMPLITUDE | F_2, 347_=21.81, P<0.001 | **<0.001** | **<0.001** | 0.21 |
| VELOCITY SLOPE | F_2, 347_=8.51, P<0.001 | 0.48 | **<0.001** | **<0.001** |
| AMPLITUDE SLOPE | F_2, 347_=34.29, P<0.001 | **<0.001** | 0.43 | **<0.001** |

**Supplementary Table 2: Comparisons of kinematic parameters between Parkinson’s disease (PD) patients belonging to Cluster 1, Cluster 2, and healthy controls (HCs).** N° MOV: number of movements, CV: coefficient of variation. P= HCs vs Cluster 1 PD, P**= HCs vs Cluster 2 PD, P***= Cluster 1 vs Cluster 2 PD. Note that the one-way analyses of variance (ANOVAs) revealed significant group effects across all kinematic variables. Post hoc comparisons revealed that for the number of movements, HCs differed from Cluster 1 but not Cluster 2, while Cluster 1 showed lower values than Cluster 2. Regarding the CV, HCs differed from Cluster 1 but not Cluster 2, with Cluster 1 exhibiting higher rhythm variability. Movement velocity was reduced in both clusters compared to HCs, with Cluster 1 showing lower values than Cluster 2. In contrast, movement amplitude differed between HCs and both clusters, but no significant difference was found between Clusters 1 and 2. For the velocity slope, HCs differed from Cluster 2 but not Cluster 1, although Cluster 1 showed a greater velocity decrement than Cluster 2. Similarly, in the amplitude slope, HCs differed from Cluster 1 but not Cluster 2, with Cluster 1 again showing a greater amplitude decrement.

|  | **Cut-off values** | **AUC** | **p** | **Sensitivity** | **Specificity** |
| --- | --- | --- | --- | --- | --- |
| N° MOV | 46.17 | 0.54 | 0.15 | 0.52 | 0.6 |
| CV | 0.11 | 0.71 | <0.001 | 0.63 | 0.7 |
| MOVEMENT VELOCITY | 952.33 | 0.80 | <0.001 | 0.77 | 0.73 |
| MOVEMENT AMPLITUDE | 46.69 | 0.70 | <0.001 | 0.68 | 0.61 |
| VELOCITY SLOPE | -6.92 | 0.59 | 0.02 | 0.68 | 0.51 |
| AMPLITUDE SLOPE | -0.17 | 0.62 | <0.001 | 0.56 | 0.60 |

**Supplementary Table 3: Kinematic cut-off values obtained from the receiver operating characteristics (ROC) curves analysis.** N° MOV: number of movements, CV: coefficient of variation, AUC: area under the curve. Movement velocity is expressed in degrees/sec, movement amplitude in degrees, velocity slope in (degree/sec)/n mov, amplitude slopes in (degree/n mov).

|  |  | **HCs [158]** | **PD OFF [192]** |
| --- | --- | --- | --- |
| Normal movement | - | 30 (19) | 6 (3.1) |
| 1 movement abnormality [HCs: 68 (43), PD OFF: 19 (9.9)] | D | 8 (5.1) | 3 (1.6) |
|  | B | 2 (1.3) | 1 (0.5) |
|  | H | 21 (13.3) | 4 (2.1) |
|  | SE | 37 (23.4) | 11 (5.7) |
| 2 movement abnormalities [HCs: 44 (27.8), PD OFF: 56 (29.2)] | D+B | 2 (1.3) | 3 (1.6) |
|  | D+H | 6 (3.8) | 1 (0.5) |
|  | D+SE | 10 (6.3) | 14 (7.3) |
|  | B+H | 16 (10.1) | 25 (13) |
|  | B+SE | 7 (4.4) | 10 (5.2) |
|  | H+SE | 3 (1.9) | 3 (1.6) |
| 3 movement abnormalities [HCs: 14 (8.9), PD OFF: 76 (39.6)] | D+B+H | 8 (5.1) | 40 (20.8) |
|  | D+B+SE | 1 (0.6) | 14 (7.3) |
|  | D+H+SE | 1 (0.6) | 2 (1.0) |
|  | B+H+SE | 4 (2.5) | 20 (10.4) |
| 4 movement abnormalities | D+B+H+SE | 2 (1.3) | 35 (18.2) |

**Supplementary** **Table 4:** **Bradykinesia features in patients with Parkinson’s disease (PD) and healthy controls (HCs).** D: dysrhythmia, B: bradykinesia (movement slowness), H: hypokinesia (low amplitude movement), SE: sequence effect (amplitude slope). The total number of subjects included in the two groups is indicated in square brackets. Percentages are in round brackets.

|  |  | **PD OFF [129]** | **PD ON [129]** |
| --- | --- | --- | --- |
| Normal movement | - | 5 (3.9) | 5 (3.9) |
| 1 movement abnormality [PD OFF: 13 (10.1); PD ON: 19 (14.7)] | D | 2 (1.6) | 2 (1.6) |
|  | B | 1 (0.8) | 1 (0.8) |
|  | H | 4 (3.1) | 6 (4.7) |
|  | SE | 6 (4.7) | 10 (7.8) |
| 2 movement abnormalities [PD OFF: 34 (26.4); PD ON: 53 (41.1)] | D+B | 1 (0.8) | 3 (2.3) |
|  | D+H | 0 (0) | 3 (2.3) |
|  | D+SE | 8 (6.2) | 19 (14.7) |
|  | B+H | 15 (11.6) | 19 (14.7) |
|  | B+SE | 7 (5.4) | 4 (3.1) |
|  | H+SE | 3 (2.3) | 5 (3.9) |
| 3 movement abnormalities [PD OFF: 50 (38.8); PD ON: 39 (30.2)] | D+B+H | 23 (17.8) | 17 (13.2) |
|  | D+B+SE | 10 (7.8) | 10 (7.8) |
|  | D+H+SE | 2 (1.6) | 4 (3.1) |
|  | B+H+SE | 15 (11.6) | 9 (7.0) |
| 4 movement abnormalities | D+B+H+SE | 27 (20.9) | 12 (9.3) |

**Supplementary Table 5:** **Bradykinesia features in patients with Parkinson’s disease (PD) patients tested after an overnight withdrawal (OFF medication state) and with their usual dopaminergic therapy (ON medication state).** D: dysrhythmia, B: bradykinesia (movement slowness), H: hypokinesia (low amplitude movement), SE: sequence effect (amplitude slope). The total number of subjects is indicated in square brackets. Percentages are in round brackets.

|  |  | **P(HCs∣Feat Combination)** | **P(PD∣Feat Combination)** |
| --- | --- | --- | --- |
| Normal movement | - | 83.3 | 16.7 |
| 1 movement abnormality [P(HCs∣Feat Count)=78.2; P(PD∣Feat Count)=21.8] | D | 72.7 | 27.2 |
|  | B | 66.7 | 33.3 |
|  | H | 84 | 16 |
|  | SE | 77.1 | 22.9 |
| 2 movement abnormalities [P(HCs∣Feat Count)=44; P(PD∣Feat Count)=56] | D+B | 40 | 60 |
|  | D+H | 85.7 | 14.3 |
|  | D+SE | 41.7 | 58.3 |
|  | B+H | 39 | 60.1 |
|  | B+SE | 21.9 | 78.1 |
|  | H+SE | 50 | 50 |
| 3 movement abnormalities [P(HCs∣Feat Count)=15.5; P(PD∣Feat Count)=84.4] | D+B+H | 16.7 | 83.3 |
|  | D+B+SE | 6.7 | 93.3 |
|  | D+H+SE | 33.3 | 66.7 |
|  | B+H+SE | 16.7 | 83.3 |
| 4 movement abnormalities | D+B+H+SE | 5.4 | 94.6 |

**Supplementary Table 6:** **Conditional probabilities of a participant belonging to either Parkinson’s disease (PD) and healthy controls (HCs) groups based on specific bradykinesia features, their combinations and number of features (feat count).** D: dysrhythmia, B: bradykinesia (movement slowness), H: hypokinesia (low amplitude movement), SE: sequence effect (amplitude slope).

|  |  | **P(PD OFF∣Feat Combination)** | **P(PD ON∣Feat Combination)** |
| --- | --- | --- | --- |
| Normal movement (p=1) | - | 50.0 | 50.0 |
| 1 movement abnormality [P(OFF∣Feat Count)=40.6; P(ON∣Feat Count)=59.4] | D | 50.0 | 50.0 |
|  | B | 50.0 | 50.0 |
|  | H | 40.0 | 60.0 |
|  | SE | 37.5 | 62.5 |
| 2 movement abnormalities [P(OFF∣Feat Count)=39.1; P(ON∣Feat Count)=60.9] | D+B | 25 | 75 |
|  | D+H | 0.0 | 100.0 |
|  | D+SE | 29.6 | 70.4 |
|  | B+H | 44.1 | 55.9 |
|  | B+SE | 63.6 | 36.4 |
|  | H+SE | 37.5 | 62.5 |
| 3 movement abnormalities [P(OFF∣Feat Count)=56.3; P(ON∣Feat Count)=43.8] | D+B+H | 57.5 | 42.5 |
|  | D+B+SE | 50.0 | 50.0 |
|  | D+H+SE | 33.3 | 66.7 |
|  | B+H+SE | 62.5 | 37.5 |
| 4 movement abnormalities | D+B+H+SE | 69.2 | 30.8 |

**Supplementary Table 7:** **Conditional probabilities of a Parkinson’s disease (PD) participant belonging to either the OFF and ON medication state based on specific bradykinesia features, their combinations and number of features (feat count).** D: dysrhythmia, B: bradykinesia (movement slowness), H: hypokinesia (low amplitude movement), SE: sequence effect (amplitude slope).

|  |  | **HCs [18]** | **PD OFF [80]** | **P(HCs∣Feat Comb)** | **P(PD∣Feat Comb)** |
| --- | --- | --- | --- | --- | --- |
| Normal movement | - | 2 (11.1) | 0 (0.0) | *100.0* | *0.0* |
| 1 movement abnormality [HCs: 7 (38.9), PD OFF: 2 (2.5); *P(HCs∣Feat Count)=77.3; P(PD∣Feat Count)=22.4]* | D | 4 (22.2) | 1 (1.3) | *79.6* | *20.4* |
|  | B | 2 (11.1) | 1 (1.3) | *66.1* | *33.9* |
|  | H | 0 (0.0) | 0 (0.0) | *-* | *-* |
|  | SE | 1 (5.6) | 0 (0.0) | *100.0* | *0.0* |
| 2 movement abnormalities [HCs: 6 (33.3), PD OFF: 15 (18.8)**;** *P(HCs∣Feat Count)=28.1; P(PD∣Feat Count)=71.9]* | D+B | 1 (5.6) | 2 (2.5) | *32.8* | *67.2* |
|  | D+H | 0 (0.0) | 0 (0.0) | *-* | *-* |
|  | D+SE | 3 (16.7) | 8 (10.0) | *26.8* | *73.2* |
|  | B+H | 1 (5.6) | 0 (0.0) | *100.0* | *0.0* |
|  | B+SE | 1 (5.6) | 5 (6.3) | *16.3* | *83.7* |
|  | H+SE | 0 (0.0) | 0 (0.0) | *-* | *-* |
| 3 movement abnormalities [HCs: 2 (11.1), PD OFF: 34 (42.5); *P(HCs∣Feat Count)=5.4; P(PD∣Feat Count)=94.6]* | D+B+H | 2 (11.1) | 20 (25.0) | *8.9* | *91.1* |
|  | D+B+SE | 0 (0.0) | 12 (15.0) | *0.0* | *100.0* |
|  | D+H+SE | 0 (0.0) | 2 (2.5) | *0.0* | *100.0* |
|  | B+H+SE | 0 (0.0) | 0 (0.0) | *-* | *-* |
| 4 movement abnormalities | D+B+H+SE | 1 (5.6) | 29 (36.3) | *3.3* | *96.7* |

**Supplementary Table 8:** **Bradykinesia features in participants belonging to Cluster 1.** PD. Parkinson’s disease, HCs: healthy controls, D: dysrhythmia, B: bradykinesia (movement slowness), H: hypokinesia (low amplitude movement), SE: sequence effect. The total number of subjects included in the two groups are indicated in square brackets. Percentages are in round brackets. Numbers in italics indicate the conditional probabilities of a participant belonging to either PD and HCs groups based on specific bradykinesia features, their combinations (feat comb) and number of features (feat count). Note that Cluster 1 included more PD patients than HCs. Compared to HCs, a higher number of patients showed abnormal movements (p=0.02 by Fisher’s exact test). Finally, the number of bradykinesia features differed between PD and HCs included in Cluster 1 (p<0.001 by Mann-Whitney U test).

|  |  | **HCs [140]** | **PD OFF [112]** | **P(HCs∣Feat Comb)** | **P(PD∣Feat Comb)** |
| --- | --- | --- | --- | --- | --- |
| Normal movement | - | 28 (20) | 6 (5.4) | *82.3* | *17.6* |
| 1 movement abnormality [HCs: 61 (43.6), PD OFF: 17 (15.2); *P(HCs∣Feat Count)=78.2; P(PD∣Feat Count)=21.8]* | D | 4 (2.9) | 2 (1.68) | *66.7* | *33.3* |
|  | B | 0 (0.0) | 0 (0.0) | *-* | *-* |
|  | H | 21 (15.0) | 4 (3.6) | *84.0* | *16.0* |
|  | SE | 36 (25.7) | 11 (9.8) | *76.6* | *23.4* |
| 2 movement abnormalities [HCs: 38 (27.1), PD OFF: 41 (36.6); *P(HCs∣Feat Count)=48.1; P(PD∣Feat Count)=51.9]* | D+B | 1 (0.7) | 1 (0.9) | *50.0* | *50.0* |
|  | D+H | 6 (4.3) | 1 (0.9) | *85.7* | *14.3* |
|  | D+SE | 7 (5.0) | 6 (5.4) | *53.8* | *46.1* |
|  | B+H | 15 (10.7) | 25 (22.3) | *37.5* | *62.5* |
|  | B+SE | 6 (4.3) | 5 (4.5) | *60.0* | *40.0* |
|  | H+SE | 3 (2.1) | 3 (2.7) | *50.0* | *50.0* |
| 3 movement abnormalities [HCs: 12 (8.6), PD OFF: 42 (37.5); *P(HCs∣Feat Count)=22.2; P(PD∣Feat Count)=77.8]* | D+B+H | 6 (4.3) | 20 (17.9) | *23.1* | *76.9* |
|  | D+B+SE | 1 (0.7) | 2 (1.8) | *33.3* | *66.7* |
|  | D+H+SE | 1 (0.7) | 0 (0.0) | *100.0* | *0.0* |
|  | B+H+SE | 4 (2.9) | 20 (17.9) | *16.7* | *83.3* |
| 4 movement abnormalities | D+B+H+SE | 1 (0.7) | 6 (5.4) | *14.3* | *85.7* |

**Supplementary Table 9:** **Bradykinesia features in participants belonging to Cluster 2.** PD. Parkinson’s disease, HCs: healthy controls, D: dysrhythmia, B: bradykinesia (movement slowness), H: hypokinesia (low amplitude movement), SE: sequence effect. The total number of subjects included in the two groups are indicated in square brackets. Percentages are in round brackets. P from Mann-Whitney U test. Significant values are shown in bold. Numbers in italics indicate the conditional probabilities of a participant belonging to either PD and HCs groups based on specific bradykinesia features, their combinations (feat comb) and number of features (feat count). In Cluster 2, we noted a roughly equal representation from the PD and HCs. Compared to HCs, a higher number of patients showed abnormal movements (p=0.005 by Fisher’s exact test). The number of bradykinesia features differed between PD and HCs included in Cluster 2 (p<0.001 by Mann-Whitney U test). Note that in Cluster 2, the combination of bradykinesia and sequence effect without additional features had no discriminatory power.

|  | **standardized β** | **t** | **p** |
| --- | --- | --- | --- |
| MDS-UPDRS III score | 0.37 | 5.27 | **<0.001** |
| Age | -0.24 | -3.33 | **0.001** |
| MoCA | -0.16 | -2.28 | **0.02** |

**Supplementary Table 10. Regression analysis conducted Parkinson’s disease (PD) patients.** Movement Disorder Society-sponsored revision of the Unified Parkinson's Disease Rating Scale, part III, LEDD: levodopa equivalent daily dose. MoCA: Montreal Cognitive Assessment. The analysis revealed a statistically significant model that explained 16.1% of the variance in the number of motor abnormalities [adjusted R²=0.148, F(3, 188)= 12.06, p<0.001]. Other variables, including sex, BDI, disease duration, levodopa equivalent daily dose (LEDD), and Hoehn & Yahr stage were excluded from the model as they did not significantly enhance its explanatory power.

|  | **standardized β** | **t** | **p** |
| --- | --- | --- | --- |
| MDS-UPDRS III score | 0.54 | 5.65 | **<0.001** |
| LEDD | -0.30 | -3.16 | **0.002** |
| Sex | 0.15 | 1.85 | 0.07 |
| Age | -0.23 | -2.68 | **0.01** |
| MoCA | -0.19 | -2.39 | **0.02** |

**Supplementary Table 11. Regression analysis conducted in the subsample of 129 Parkinson’s disease (PD) patients tested in the ON and OFF medication state.** Movement Disorder Society-sponsored revision of the Unified Parkinson's Disease Rating Scale, part III, LEDD: levodopa equivalent daily dose. MoCA: Montreal Cognitive Assessment. The analysis revealed a statistically significant model that explained 24.4% of the variance in the number of motor abnormalities [adjusted R² = 0.244, F(5, 123) = 9.28, p<0.001]. Other variables, including disease duration, Beck Depression Inventory (BDI) scores, and delta MDS-UPDRS III were excluded from the model as they did not significantly enhance its explanatory power.


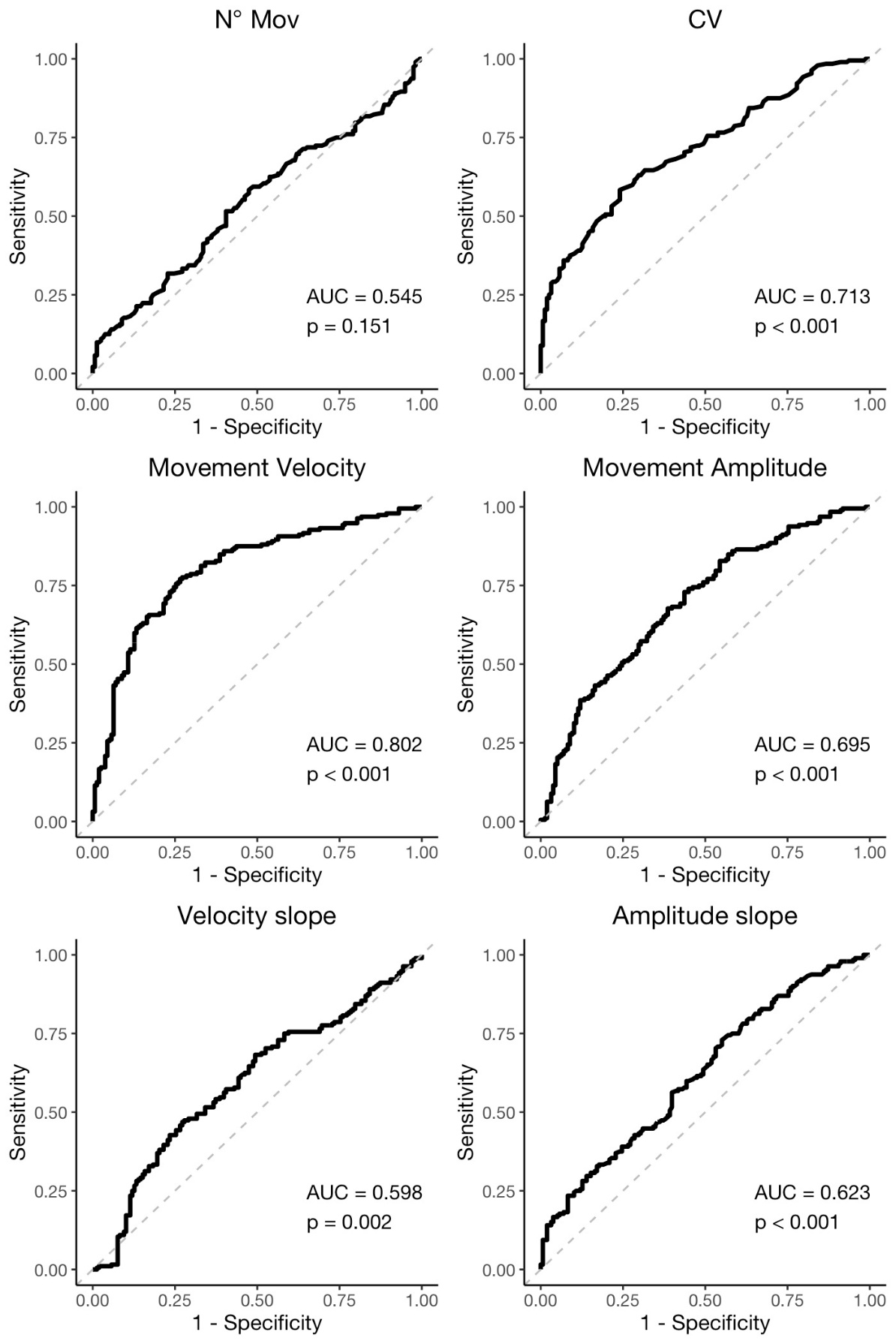


**Supplementary Figure 1:** Receiver operating characteristic (ROC) curves. ROC curves were used to graphically represent the diagnostic properties of the finger-tapping kinematic variables (number of movements, i.e., N° MOV, coefficient of variation, i.e., CV, movement velocity and amplitude, velocity and amplitude slope, i.e., sequence effect). The value of area under the ROC curve (AUC) measures how well the model can discriminate between subjects. To determine the optimal cut-off point, we employed the ‘Closest-to-(0,1) Method’, which minimizes the Euclidean distance between the ROC curve and the ideal point (0,1) on the ROC plane. This method optimally balances sensitivity (true positive rate) and specificity (true negative rate) for classification.


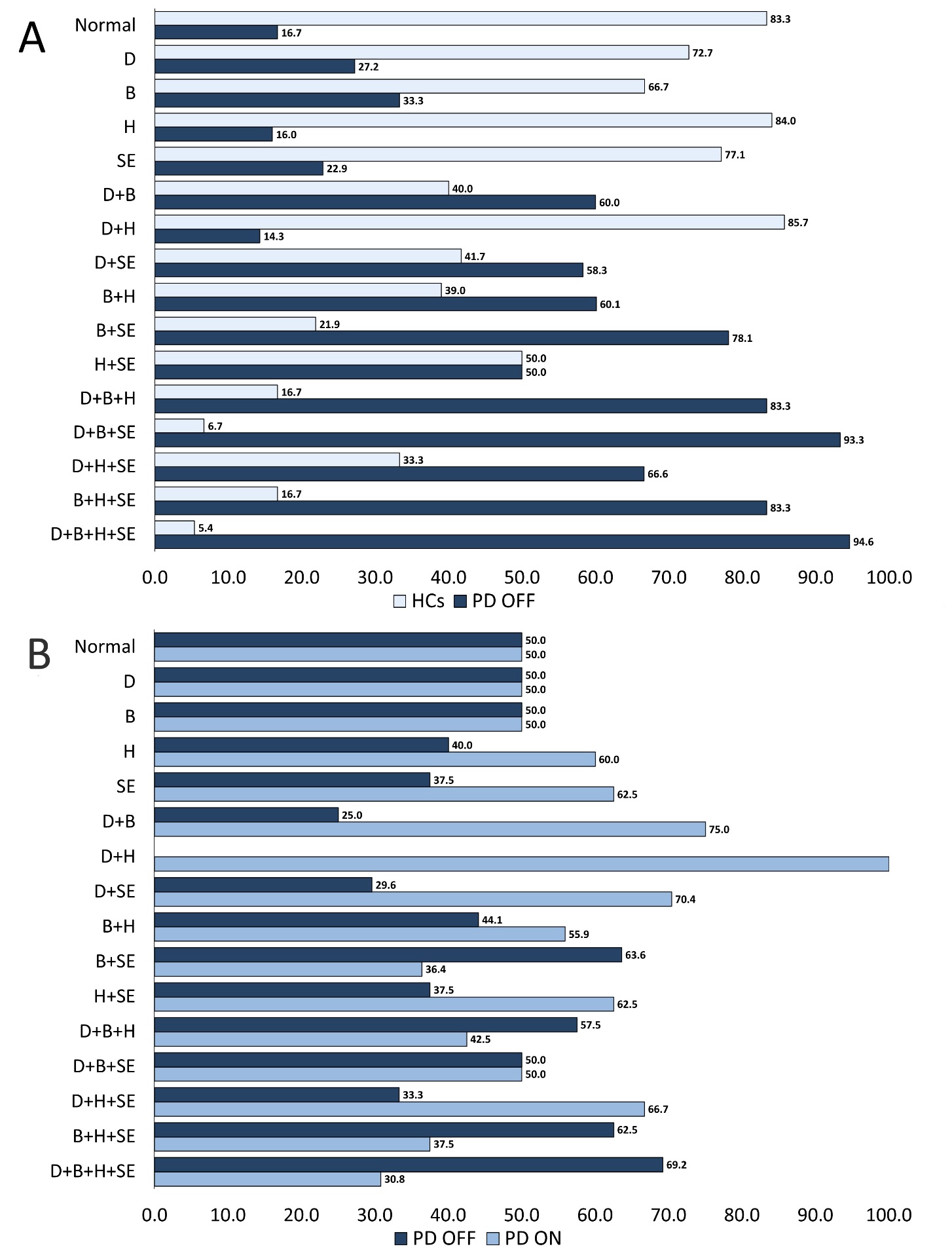


**Supplementary Figure 2: Conditional probabilities.** We calculated the conditional probabilities of a participant belonging to (A) either Parkinson’s disease (PD) and healthy controls (HCs) groups or (B) OFF and ON medication state based on specific bradykinesia features and combinations.
